# Externalizing behavior is prospectively associated with intake of added sugar and sodium among low socioeconomic status preschoolers in a sex-specific manner

**DOI:** 10.1186/s12966-017-0591-y

**Published:** 2017-10-03

**Authors:** Erica C. Jansen, Alison L. Miller, Julie C. Lumeng, Niko Kaciroti, Holly E. Brophy Herb, Mildred A. Horodynski, Dawn Contreras, Karen E. Peterson

**Affiliations:** 1 Department of Nutritional Sciences, School of Public Health, Ann Arbor, MI USA; 2 Center for Human Growth and Development, University of Michigan, Ann Arbor, MI USA; 3 Department of Health Behavior and Health Education, School of Public Health, Ann Arbor, MI USA; 4 Department of Pediatrics, University of Michigan, Ann Arbor, MI USA; 5 Department of Human Development and Family Studies, Michigan State University, East Lansing, MI USA; 6 College of Nursing, Michigan State University, East Lansing, MI USA; 7 Health and Nutrition Institute, Michigan State University Extension, East Lansing, MI USA; 8 Department of Nutritional Sciences, University of Michigan School of Public Health, 1415 Washington Heights, Ann Arbor, MI 48109 USA

**Keywords:** Sodium, Added sugar, Externalizing behavior

## Abstract

**Background:**

High intake of added sugar and sodium is a public health concern for preschool-aged children living in the US. Externalizing behavior may predict higher consumption of added sugar and/or sodium; however, previous studies have mostly been cross-sectional. The aim was to evaluate whether externalizing behavior is prospectively related to added sugar and intake in a sex-specific manner among preschoolers.

**Methods:**

This was a secondary analysis of 524 preschool children (48% male) from Michigan who participated in an obesity prevention trial that occurred during one school year from 2011 to 2015. Teacher-assessed externalizing behaviors and three 24-h dietary recalls were completed at baseline and follow-up. We used linear mixed effects regression to evaluate the association between externalizing behavior at baseline and added sugar (% of total Calories) and sodium intake (mg/1000 Calories) at follow-up. In adjusted analysis, we included baseline income-to-needs ratio, child race/ethnicity, and baseline overweight status. All models were adjusted for total energy intake and accounted for clustering by classroom.

**Results:**

Baseline externalizing behavior was positively associated with added sugar intake at follow-up among boys; after adjustment for confounders, every 5 points lower externalizing T-score (corresponding to higher externalizing behavior) was associated with a 0.6 higher percentage of added sugar per total Calories (95% CI 0.2 to 1.1; *P* value = 0.004). In contrast, girls with higher levels of externalizing behavior had lower consumption of added sugars; after confounder adjustment, every 5 points lower externalizing T-score was related to 0.6 lower percentage intake (95% CI -1.0 to −0.1; *P* value = 0.01). Baseline externalizing behavior was inversely associated with sodium intake at follow-up among boys. After potential confounder adjustment, for every 5 points lower externalizing behavior T-score, there was a 22 mg/1000 Cal lower sodium intake (95% CI -45 to 1; *P* value = 0.06). In contrast, after adjustment for confounders, every 5 points lower externalizing T-score among girls was related to 24 mg/1000 Cal higher sodium intake (95% CI 1 to 46; *P* value = 0.04).

**Conclusions:**

Externalizing behavior among preschool-aged children was prospectively related to added sugar and sodium intake in a sex-dependent manner.

**Trial registration:**

NCT01398358 Registered 19 July 2011.

**Electronic supplementary material:**

The online version of this article (10.1186/s12966-017-0591-y) contains supplementary material, which is available to authorized users.

## Background

High intake of added sugar and sodium among US preschool-aged children is a public health concern; recent nationally-representative surveys indicate that US preschool-aged children on average obtain between 13 and 16% of total Calories from added sugar [1] and over 90% of children ages 4–8 years (y) old exceed recommended levels of sodium intake [2] This is concerning because high consumption of sodium as well as added sugar in early childhood has been implicated in the development of cardiometabolic risk factors during childhood and adolescence [3, 4]. In addition, there is evidence that free sugar intake >10% of total Calories is positively associated with dental caries among children [5]. Thus, elucidating the predictors of higher added sugar and sodium intake among young children could provide valuable information for intervention.

One potential predictor of these dietary risk factors is externalizing behavior. Externalizing behavior during early childhood is marked by angry, aggressive, selfish and oppositional conduct; it includes fighting with or bullying other children, physically hitting, and taking things from others [6]. Externalizing behaviors have been linked with higher consumption of salty and/or sugary foods in a few cross-sectional studies [7, 8]. For example, a study of 13,486 Iranian children and adolescents found that those with higher self-reported aggressive behaviors also reported higher consumption of salty snacks [7]. Another study among 1324 adolescents in Australia found that those with more externalizing behaviors had higher intake of fast foods, confectionary items, and red meat [8]. There has also been one longitudinal study examining externalizing behavior and frequency of sugary food consumption, although not sodium. In this study of 6997 Norwegian children, researchers found that children with higher externalizing behavior at age 1.5 y had higher intake of sugary foods at age 3 y and 7 y [9]. A longitudinal study design is important as it separates the exposure from the outcome in time, lending stronger support to the notion that behavior leads to consumption patterns. There are some plausible mechanisms to support this pathway. For example, externalizing behavior during early childhood could be a marker of stress [10], and stress has been linked to higher consumption of snacks and sweet foods in young children [11–13]. Also, children with more externalizing behavior may have greater control over dietary choices because caregivers impose fewer restrictions and/or use food to calm them [14, 15].

The aim of this study was to examine the longitudinal association between externalizing behavior and intake of added sugar (% of Calories) and sodium (mg/1000 Calories) among low socioeconomic status (SES) preschoolers [16]. We hypothesized that children with higher levels of externalizing behavior at baseline would have higher percentage of Calories from added sugar intake and sodium intake (mg/1000 Calories) at follow-up. All analyses were stratified by sex, a potential effect modifier when examining associations between behavior and diet [13].

## Methods

### Study population

The present study was a secondary data analysis of the Growing Healthy Study, a cluster-randomized community-based obesity intervention trial among 697 preschoolers in Head Start programs in urban and rural Michigan from 2011 to 2015. The study is described in detail elsewhere [16]. Briefly, children in 18 classrooms per year were randomized to one of three study arms. Parents/guardians were invited to participate and received up to $150 for data collection activities. One arm consisted of exposure to the Preschool Obesity Prevention Series (POPS), a Social Cognitive Theory-based approach to obesity prevention. The second arm included POPS exposure in addition to the Incredible Years Series, a program that emphasizes positive behavioral management techniques [17]. The interventions consisted of classroom lessons for children during Head Start as well as separate parent/guardian lessons. The control arm consisted of usual Head Start exposure. Baseline visits occurred during the fall of one school year and follow-up visits occurred during the following spring, with a mean duration of 6 months (standard deviation 1 month). Exclusion criteria included the child having significant developmental disability, the child being in foster care, or the parent/guardian not being fluent in English. Informed consent was obtained, and the study was approved by the Institutional Review Boards of the University of Michigan and Michigan State University.

### Data collection

#### Externalizing behavior

Externalizing behaviors were assessed by Head Start teachers, who completed the Social Competence and Behavior Scale (SCBE) during baseline and follow-up visits [6]. This 60-item instrument has been validated for children aged 2.5 to 6 y and is used to assess child social, emotional and behavioral functioning. Teachers were asked to rate aspects of the child’s behavior on a 6-point Likert scale (“never” to “always”). The 15-item Externalizing Problems subscale assessed externalizing behaviors (e.g., “gets angry when interrupted”; “refuses to share toys”; Cronbach’s alpha = 0.92). Items are summed and typically scored such that higher scores indicate lower levels of externalizing behavior [6]. T- scores standardized for age and sex were used, which typically have a mean of 50 and standard deviation of 10. Scores were examined as a continuous variable, and beta estimates were reported per 5-points lower externalizing T-scores (corresponding to higher externalizing behavior).

#### Diet measures

Multiple 24-h dietary recalls were used to measure usual dietary consumption of the child at follow-up (including supplement intake). Trained dieticians sought to collect three unannounced 24-h dietary recalls via phone from parents/guardians, who were provided handouts showing child-appropriate portion sizes. The United States Department of Agriculture (USDA) 5-step automated multiple-pass method, which has been shown to improve accuracy of diet recall [18] was used. Calls occurred over a 2–3 week period, and parents/guardians were asked to provide 24-h recalls from 2 weekdays and 1 weekend day. Dieticians contacted the same parent/guardian each time and confirmed this parent/guardian had knowledge regarding the child’s consumption that day. Because the children consume food at Head Start classrooms during school days, research staff observed and recorded what the child ate during the same weekdays that the parents/guardians provided 24-h recall information. Nutrient and caloric content of each food item was calculated with the aid of the USDA Food Composition Database using the Nutrition Data System for Research program (NDSR, University of Minnesota, Minneapolis, Minnesota). Calories of all foods consumed in the 24-h period were summed to calculate total energy intake. Recalls with implausible energy intakes were removed prior to analysis (*n* = 20 of 1287 total recalls at follow-up) using established criteria [19]. To reduce within-person random measurement error, summary variables representing nutrient contents per day for each participant were created by averaging all the valid 24-h recalls collected at follow-up. Added sugar was analyzed as average percentage of total Calories and sodium intake was analyzed as average milligrams (mg) per 1000 Calories. Nutrients were also classified into dichotomous variables for descriptive statistics, based on recommended cutoffs of intake in the United States (US) [20]. Higher than recommended intake for added sugar was classified as ≥10% of Calories from added sugar; for sodium, higher than recommended intake for sodium was classified as ≥2300 mg/day.

#### Other covariates

Trained research assistants measured height (in meters) and weight (in kilograms) of children and parents/guardians during home visits using calibrated equipment (Seca 213/217 portable stadiometer and Detecto Portable Scale Model #DR550C, respectively). BMI-for-age percentiles were calculated with the use of the Center for Disease Control (CDC) reference [21], and overweight/obesity was defined as ≥85th percentile. As a part of the demographic questionnaire administered during the first visit, parents/guardians provided information on the child’s race/ethnicity, parent/guardian’s education, and household income from all sources. Child race/ethnicity was classified into two categories: non-Hispanic White and Hispanic or not White. Education was categorized into those parent/guardians who did not graduate from high school, those who graduated from high school/obtained a General Educational Development (GED) certification, and those with a post-high school education. Income-to-needs ratio was calculated as the total reported household income divided by the federal poverty line for a family of the same size during the corresponding year, and it was categorized into quartiles. Parents/guardians also answered questions about their child’s physical activity (hours per week) and screen time (hours per day) habits. Screen time and physical activity were each categorized into 4 categories, as shown in Table 2. Finally, parents/guardians completed the Center for Epidemiologic Studies Depression (CES-D) scale, a 20-item survey with higher scores indicating more depression symptoms [22]. Depression symptoms were classified as a dichotomous variable, with those reporting a score of ≥16 considered to have clinically significant depressive symptoms [23].

### Data analysis

There were 697 children enrolled in the trial. Thirty-seven children did not have teacher-reported SCBE measures at baseline; of these, three children were excluded because none of the collected recalls were deemed valid, and 133 were excluded because no dietary information was provided. Thus, the analytic sample included 524 children. The parents/guardians who took part in the study were mostly biological mothers (90%), some biological fathers (4%) or other relatives (4%), and a few were foster parents (1%), or other (1%). The analytic sample did not differ from the baseline study population according to the sociodemographic characteristics listed in Table 1. All analyses were stratified by sex. This decision was made a priori, and sex differences were confirmed in preliminary interaction models (*P* < 0.05 for sodium intake model and *P* < 0.1 for added sugar models).

**Table 1:** Characteristics of the study population

Baseline child and household correlates	Mean ± SD (unless otherwise specified)
Child’s age, months	49.5 ± 6.2
Male sex, %	48
Overweight/obese, %	34
Physical activity, hours/wk^a^	18.5 ± 10.9
Screen time, hours/day^b^	2.6 ± 1.6
Added sugar intake, % of total Calories^c^	12.9 ± 6.1
Sodium intake, mg/1000 Calories^d^	1604 ± 332
Externalizing SCBE T-score^e^	51.9 ± 9.4
Parent/guardian education level, %	
Did not graduate HS	15
HS graduate/GED	33
Post-HS education	52
Household income-to-needs ratio^f^	0.86 ± 0.54
Parent/guardian CES-D score^g^	
≥ 16, depressed, %	30
Child race/ethnicity	
White, non-Hispanic, %	51

^a^There are not consistent physical activity recommendations for children <6 in the US, although Canadian and Australian recommendations are 3 h/day (21 h/week) [40]

^b^Recommendations for children under 5 years are <2 h/day [41]

^c^US dietary recommendations are <10% of total Caloric intake from added sugar per day [20]

^d^US dietary recommendations are <2300 mg per day [20]; depending on Calorie needs which range from 1200 to 2000 Calories in this age group, that equates to >1150 mg/1000 Calories to >1917 mg/1000 Calories

^e^An externalizing SCBE T-score of 70 is considered the cutoff for externalizing behavior [6]

^f^An income-to-needs poverty ratio < 2 is classified as poverty [42, 43]

^g^The CES-D scores range from 0 to 60, with a score of 16 or greater considered at risk of clinical depression [22]

First, the mean ± standard deviation (SD) externalizing behavior T-scores at baseline according to baseline child and household covariates were examined. The mean ± SD percentage of Calories from added sugar intake and sodium (mg/1000 Calories) at follow-up according to baseline child and household covariates were also examined. The *P* values of unadjusted associations were obtained using linear mixed effects regression models with the nutrient intake or behavior scores as the outcome and the baseline covariate as the predictor. Clustering by classroom was accounted for in all models by using a random intercept term. To determine the primary food sources of sodium and added sugar intake, the total amount of sodium and added sugar was analyzed at the food group (specified by the NDSR software) level. The sodium and added sugar content from every food item consumed (including all children and both time points) was summed and then the percentage of sodium or added sugar contributed by each food group (specified by NDSR) was calculated. The top 10 food group sources of each nutrient were ranked according to percentage contributed.

To evaluate the crude association between baseline externalizing behavior and continuous percentage of Calories from added sugar or mg of sodium/1000 Calories at follow-up, linear mixed effects regression models were utilized, with the nutrient as the outcome and continuous externalizing behavior T-score as the exposure. A random intercept for classroom was used to account for clustering (since children in the same classroom may be more similar in terms of diet and behavior), and total energy intake at follow-up was accounted for.

In adjusted analyses, models included indicator variables for child race/ethnicity, quartiles of income-to-needs ratio, and a dichotomous variable for overweight/obese status. These covariates were chosen based on prior knowledge. Intervention arm was not treated as a potential confounder because the exposure (baseline externalizing behavior score) was assumed to be equally distributed among intervention arms. We evaluated this assumption in sensitivity analyses where we adjusted for intervention arm. All analyses were performed in Stata 14.0.

## Results

The mean age ± SD of the children at baseline was 4.1 ± 0.5 y; 48% were boys (Table 1).

The mean standardized externalizing score ± SD for boys at baseline 51.7 ± 9.4, and for girls it was 52.2 ± 9.5. Girls who were overweight/obese and from households with lower income-to-needs ratio at baseline had higher levels of baseline externalizing behavior (Table 2).

**Table 2:** Mean ± SD externalizing SCBE T-scores^a^ at baseline, according to baseline correlates

	Boys		Girls	
Baseline child and household correlates	N	Externalizing SCBE T-score, mean ± SD	N	Externalizing SCBE T-score, mean ± SD
Child’s age				
3 y	92	51.2 ± 9.2	99	52.1 ± 9.9
4 y or older	156	52.0 ± 9.6	171	52.2 ± 9.2
*P* value^b^		0.40		0.80
Child’s race/ethnicity				
White, non-Hispanic	126	52.3 ± 8.3	139	51.3 ± 8.8
Hispanic or not White	123	51.1 ± 10.4	132	53.2 ± 10.1
*P* value		0.26		0.16
Overweight/obese status				
Not overweight/obese	150	52.5 ± 9.5	194	53.3 ± 9.2
Overweight/obese	98	50.5 ± 9.2	76	49.4 ± 9.7
*P* value		0.11		0.002
Physical activity, hours/wk				
< 7	28	52.1 ± 11.1	26	54.4 ± 8.5
7 to <14	54	50.3 ± 8.9	70	51.4 ± 10.2
14 to <24	74	52.6 ± 9.7	79	51.4 ± 8.6
≥ 24	73	52.2 ± 8.9	63	52.3 ± 10.3
*P* value		0.94		0.84
Screen time, hours/day				
< 1.5	53	51.8 ± 10.1	62	51.2 ± 9.7
1.5 to <2.5	66	52.2 ± 8.7	82	52.0 ± 9.5
2.5 to <3.5	57	51.8 ± 9.8	56	51.1 ± 9.5
≥ 3.5	56	51.4 ± 9.4	47	54.3 ± 9.5
*P* value		0.66		0.13
Parent/guardian education level				
Did not graduate HS	36	53.8 ± 9.1	42	54.6 ± 9.8
HS graduate/GED	81	51.7 ± 9.4	92	50.2 ± 9.4
Post-HS education	134	51.1 ± 9.5	135	52.8 ± 9.1
*P* value		0.45		0.50
Household income-to-needs ratio				
Quartile 1, <0.47	46	53.5 ± 10.5	66	49.6 ± 9.0
Quartile 2, ≥0.47 to <0.76	55	51.6 ± 10.3	58	54.3 ± 8.8
Quartile 3, ≥0.76 to <1.12	56	52.2 ± 9.2	55	52.5 ± 10.8
Quartile 4, ≥1.12	52	51.4 ± 8.3	55	53.2 ± 9.4
*P* value		0.91		0.05
Parent/guardian CES-D score				
< 16, not depressed	168	51.9 ± 9.6	163	52.5 ± 9.5
≥ 16, depressed	65	51.6 ± 9.3	80	51.1 ± 9.8
*P* value		0.35		0.34

^a^Higher T-scores on the SCBE indicate lower levels of externalizing behavior

^b^From a linear mixed effects model with externalizing SCBE T-score as the outcome and a continuous variable for each ordinal sociodemographic predictor. For nominal categorical variables, a type III F test was used. A random intercept was specified to account for clustering by classroom

The mean percentage of Calories from added sugar ± SD at follow-up was 12.9 ± 5.9 among boys; it was 12.8 ± 6.3 among girls. The mean sodium intake ± SD (mg/1000 Calories) for boys at follow-up was 1596 ± 333. For girls it was 1610 ± 332 mg/1000 Calories. Sixty-nine percent of boys and 65% of girls consumed higher than recommended intake from added sugar (≥ 10% of total Calories) at follow-up. Fifty-one percent of boys and 47% of girls had higher than recommended average sodium intakes (≥ 3200 mg/day) at follow-up. The highest food source of added sugar in this population was soda, whereas meat, poultry, and fish recipes were the highest source of sodium (Additional file 1: Table S1). In both boys and girls, higher levels of parent/guardian-reported screen time were associated with a higher percentage of Calories from added sugar at follow-up (Table 3). For boys, those whose mothers had a CESD score ≥ 16 had a higher % of Calories from added sugar at follow-up. White, non-Hispanic children had a lower average intake of mg sodium/1000 Calories at follow-up than children of other race/ethnicities (Table 3). Among girls only, being overweight/obese was associated with higher mg sodium/1000 Calories at follow-up than the referent group.

**Table 3:** Mean ± SD added sugar and sodium intake at follow-up according to baseline correlates

		Boys		Girls		
Baseline child and household correlates	N	Added sugar, % of total Calories	Sodium, mg/1000 Calories	N	Added sugar, % of total Calories	Sodium, mg/1000 Calories
Child’s age						
3 y	92	12.5 ± 7.3	1586 ± 375	99	12.6 ± 6.5	1601 ± 326
4 y or older	156	13.2 ± 5.0	1599 ± 308	171	13.0 ± 6.1	1614 ± 336
*P* value^1^		0.39	0.99		0.16	0.55
Child’s race/ethnicity						
White, non-Hispanic	126	13.5 ± 6.0	1539 ± 308	139	12.4 ± 6.3	1562 ± 313
Hispanic or not White	123	12.3 ± 5.8	1661 ± 334	132	13.5 ± 6.2	1663 ± 347
*P* value		0.07	0.003		0.43	0.02
Overweight/obese status						
Not overweight/obese	150	12.7 ± 5.5	1569 ± 345	194	12.8 ± 6.0	1587 ± 314
Overweight/obese	98	13.2 ± 6.5	1632 ± 315	76	13.1 ± 7.0	1666 ± 370
*P* value		0.66	0.12		0.78	0.03
Physical activity, hours/wk						
< 7	28	12.9 ± 5.5	1598 ± 301	26	13.6 ± 6.4	1609 ± 378
7 to <14	54	12.6 ± 5.5	1601 ± 324	70	13.1 ± 7.0	1625 ± 362
14 to <24	74	13.1 ± 5.8	1617 ± 328	79	12.7 ± 5.6	1651 ± 310
≥ 24	73	13.2 ± 6.6	1588 ± 376	63	12.4 ± 5.9	1598 ± 323
*P* value		0.56	0.70		0.61	0.67
Screen time, hours/day						
< 1.5	53	12.3 ± 4.3	1548 ± 313	62	11.6 ± 5.3	1576 ± 319
1.5 to <2.5	66	12.2 ± 5.7	1627 ± 308	82	13.1 ± 5.6	1679 ± 363
2.5 to <3.5	57	13.0 ± 5.4	1601 ± 424	56	13.2 ± 6.0	1616 ± 295
≥ 3.5	56	15.0 ± 7.7	1629 ± 284	47	14.1 ± 8.1	1610 ± 324
*P* value		0.01	0.28		0.01	0.81
Parent/guardian education level						
Did not graduate HS	36	13.2 ± 7.9	1623 ± 333	42	13.2 ± 7.3	1591 ± 355
HS graduate/GED	81	12.4 ± 5.6	1644 ± 318	92	12.5 ± 6.3	1626 ± 356
Post-HS education	134	13.2 ± 5.5	1560 ± 341	135	13.1 ± 6.0	1608 ± 313
*P* value		0.81	0.11		0.60	0.55
Household income-to-needs ratio						
Quartile 1, <0.47	46	12.0 ± 5.5	1587 ± 318	66	12.3 ± 5.9	1675 ± 311
Quartile 2, ≥0.47 to <0.76	55	13.4 ± 5.0	1606 ± 405	58	14.6 ± 7.6	1629 ± 333
Quartile 3, ≥0.76 to <1.12	56	13.8 ± 7.4	1631 ± 316	55	13.1 ± 6.1	1557 ± 336
Quartile 4, ≥1.12	52	13.8 ± 6.1	1583 ± 305	55	12.1 ± 5.3	1599 ± 333
*P* value		0.46	0.41		0.94	0.07
Parent/guardian CES-D score						
< 16, not depressed	168	12.5 ± 4.9	1609 ± 344	163	13.0 ± 6.0	1597 ± 305
≥ 16, depressed	65	14.6 ± 7.6	1588 ± 317	80	12.9 ± 6.8	1688 ± 365
*P* value		<0.0001	0.14		0.47	0.07

^1^Each *P* value is from a separate linear mixed effects models with added sugar (% of Calorie) or sodium (mg/1000 Calories) as the outcome, the continuous variable (for ordinal characteristics) as the exposure, and total energy intake at follow-up. For nominal variables, a type III F test was used. A random intercept was specified to account for clustering by classroom

There was a positive association between externalizing behavior at baseline and added sugar intake at follow-up among boys (Table 4). After adjustment for potential confounders, every 5 points lower externalizing T-score (more externalizing behavior) was associated with a 0.6 higher percentage of added sugar per total Calories (95% CI 0.2 to 1.1; *P* value = 0.008). In contrast, girls with higher levels of externalizing behavior had lower consumption of added sugars; after potential confounder adjustment, every 5 points lower externalizing T-score was related to 0.6 lower percentage intake (95% CI -1.0 to −0.1; *P* value = 0.01).

**Table 4:** Association between externalizing SCBE T-scores at baseline and added sugar and sodium intake at follow-up

Baseline externalizing SCBE T-scores^a^	Added sugar intake, % of Calories		Sodium intake, mg/1000 Calories	
	Unadjusted beta (95% CI)^b^	Adjusted beta (95% CI)^b,c^	Unadjusted beta (95% CI)^b^	Adjusted beta (95% CI)^b,c^
Boys				
Per 5-points lower T-score (more externalizing behaviors)	0.5 (0.1, 0.9)	0.6 (0.2, 1.1)	−19 (−41, 3)	−22 (−45, 1)
*P* value	0.008	0.004	0.09	0.06
Girls				
Per 5-point lower T-score (more externalizing behaviors)	−0.5 (−0.9, −0.1)	−0.6 (−1.0, −0.1)	28 (8, 49)	24 (1, 46)
*P* value	0.03	0.01	0.007	0.04

^a^Higher number of externalizing behaviors is equivalent to a lower T-score

^b^From a linear mixed effects regression model with continuous added sugar (% of Calories) or sodium intake (mg/1000 Calories) as the outcome and continuous externalizing SCBE T-scores as the predictor, expressed per 5 points. The model also accounted for total energy intake at follow-up. A random intercept was specified to account for clustering by classroom

^c^Adjusted for race/ethnicity, quartiles of income-to-needs ratio, and overweight/obese status at baseline as potential confounders

There was not a statistically significant association between externalizing behavior and mg sodium/1000 Calories among boys (Table 4). However, after adjustment for potential confounding, there was a marginally statistically significant inverse association between externalizing behavior at baseline and sodium intake at follow up. For every 5 points lower externalizing T-score, there was a 22 mg/1000 Calories lower sodium intake at follow-up (95% CI -45 to 1; *P* value = 0.06). In contrast, there was a positive association between externalizing behavior at baseline and sodium intake at follow-up among girls. In adjusted analysis, every 5 points lower externalizing T-score was related to 24 mg/1000 Calories greater sodium intake at follow-up (95% CI 1 to 46; *P* value = 0.04). Further adjustment for intervention arm did not alter the estimates in any of the models.

## Discussion

In this group of low-SES Michigan preschoolers, we found that preschool boys with higher levels of externalizing behavior had higher percentage of intake from added sugar but lower mg sodium/1000 Calories (after confounder adjustment). In contrast, girls with higher levels of externalizing behavior in the fall of one school year had higher intake of sodium/1000 Calories but lower intake of added sugar as a percentage of total Calories during the spring of the same school year.

Our study findings are in line with some other studies, although the sex-dependency of the findings is distinct. Our findings among boys are in agreement with those of a longitudinal study among Norwegian preschoolers [9]. This study showed that children with higher externalizing behavior (based on the Child Behavior Checklist) at 1.5 y of age were more likely to consume sweet drinks and sweet foods daily at 7 y of age. However, this study did not report on sodium intake, and it is also unclear whether the association was sex-specific. Other studies that specifically examined externalizing behaviors in relation to diet were cross-sectional. One study among 453 Belgian children between 5 and 12 y of age found a positive association between parent-reported “problem” behaviors (including emotional problems, conduct problems, hyperactivity problems, peer problems, and antisocial behavior) and sweet and fatty food consumption [12]. Another study among Iranian adolescents found that children with higher self-reported violent behaviors also had higher consumption of salty snacks [7]. There was no association between violent behaviors and sweet food consumption, but it could have been masked by not stratifying on sex. Finally, a cross-sectional population-based survey among Nova Scotian 5th graders found that those who had higher levels of self-reported externalizing behaviors had lower diet quality scores [24]. Lower diet quality often is related to high sodium and/or added sugar levels, although the study did not examine particular nutrients.

Our findings are also in agreement with some of the literature on stress and dietary patterns. For example, one study among 4th through 6th graders from rural Texas found that those with higher perceived stress also reported greater frequency of consumption of salty snacks or high sugar foods [25]. An experimental study among 43 US children aged 5–9 y of age found that greater stress-related cortisol was related to greater energy intake in the absence of hunger among the 8–9 year olds, although not in the younger children [26]. Finally, another US study showed that high stress self-efficacy (i.e., ability to handle stress) among fourth-graders was related to higher fruit and vegetable intake [27]. In summation, it is possible that the associations we observed between externalizing behavior and diet could be related to stress and emotional eating. This is plausible, given that children with externalizing behavior may also have higher levels of stress, anxiety and/or depressive symptoms [28, 29]. However, the lack of specificity of foods in prior studies precludes in-depth comparisons. In several of the studies on stress and eating, “comfort foods” including sugary, salty, and energy-dense foods, were assumed to be eaten together and were not further disentangled into sodium and added sugar intake. Prior assumptions about “comfort foods” are problematic because they may not hold in every population. In addition, it is important to separate added sugar from sodium because high intake of sodium and high intake of added sugar may have different health consequences that should be monitored accordingly (e.g. dental caries for added sugar and high blood pressure for sodium). Finally, one of the limitations in most prior studies was the lack of stratification by sex, so associations in the opposite directions could have been hidden.

There are a few potential mechanisms to explain the findings specifically related to externalizing behavior. One is that children with externalizing behaviors exhibit less effortful control and higher impulsivity [30], and higher impulsivity has been related to eating in the absence of hunger [31]. It could be that girls with externalizing behavior eat foods with higher sodium content in the absence of hunger while boys with externalizing behavior eat foods (or consume drinks) with higher added sugar in the absence of hunger. The notion that boys with externalizing behavior may prefer sweet foods is supported by a few adult studies. For example, in one small laboratory study among adults, men who were put through an acute stressor test reported a higher appetite for sweet foods than the control group, but there was a null association for women [32]. In another US study among young men, after watching a violent video game, there was a tendency towards sweet food consumption, but not salty, savory, or fatty food [33]. One possible physiological explanation for the association between aggression and higher sugar consumption is that glucose is required in order for the brain to control aggressive impulses [34]. In support, an experimental study among US undergraduates showed that those who consumed a glucose beverage immediately before being provoked showed reduced aggression compared to those who consumed a placebo drink, and this was most evident among those who scored higher on a pre-experiment aggressive trait survey. Although the authors did not conduct stratified analysis, males had higher aggressive trait scores than females. In contrast, there may be an explanation for sodium preference among females with externalizing behavior. It has been posited that because the renin-angiotensin-aldosterone system is involved in both sodium regulation and the stress response, sodium intake may help to alleviate the short-term effects of psychosocial stress [35]. Furthermore, this association is thought to be most evident among females because their average absolute salt intake is lower than males’. The main caveat to these potential mechanisms is the fact that the research is mostly from adult population and may not be generalizable to preschool-aged children.

Another possible explanation of the study findings is that parent/guardians of externalizing children may try to pacify them with highly-palatable foods [36], which are likely to contain high amounts of added sodium and/or sugar. One reason for the sex difference is that girls could be fed differently than boys due to societal expectations. For example, parents may feel more pressure to shield their daughters from gaining weight than their sons; thus, they may offer foods high in sodium (although not necessarily snack foods) rather than highly sugary foods to daughters due to the perception that sugar causes weight gain. The finding of lower percentage of added sugar intake among girls with higher externalizing behaviors supports this notion. Furthermore, given that the most common sources of sodium consumption were meat dishes, it is possible that girls with externalizing behavior did not have greater salty snack consumption, but rather ate higher proportions of salty entrees at lunch or dinner. Future studies on behavior and diet should consider sex as a potential effect modifier to evaluate the reproducibility of our findings and to further elucidate the potential mechanisms, especially among young children. Understanding mechanisms involved in these associations could inform interventions designed to reduce added sugar and sodium intake among children with externalizing behavior problems. Although parents/guardians would likely be the target of these interventions, teachers may be able to recognize externalizing behaviors that parents/guardians do not perceive; and thus could play a vital role in educating parents/guardians. Future studies investigating the food consumed at school versus home could further elucidate the role that teachers and the school environment could play in establishing healthy eating habits.

Although there were clear differences in added sugar and sodium intake according to externalizing behavior and sex, only about one-third of the children met added sugar recommendations and one-half met sodium guidelines. This is concerning, because the dietary habits and preferences established early in life tend to track into adulthood [37]. Furthermore, both added sugar and sodium intake during childhood are related to adverse health outcomes, even in early life [3, 4]. Thus, although the children with externalizing behavior problems were more likely to consume either added sugar or sodium, the entire population could benefit from reductions in sodium and added sugar in the food supply. This could be achieved by programs such as the National Salt Reduction Initiative, a US program which calls for voluntary reductions in sodium content of packaged foods and restaurants [38]. Policy-level recommendations for reducing added sugar content of products should also be considered. At the parent/guardian level, providing more specific information on specific foods to avoid or limit to reduce consumption of added sugar or sodium could prove useful. This is more pertinent for sodium, as many public health campaigns currently focus on sugar consumption.

This study has strengths and limitations. One strength was the repeated measures of both diet and behavior, which allowed us to examine the directions using longitudinal analysis. The prospective design was also important for reducing potential recall bias, because the behavior of the child at baseline most likely did not affect the perception of diet at follow-up and vice versa. Another was the incorporation of classroom as a clustering variable in our models; this ensures our standard errors were valid. Another strength was that diet was reported by evaluators unaware of the externalizing behavior score of the child. The fact that the evaluators of externalizing behavior were teachers may increase the objectivity of these scores. There were also limitations. We had to rely on multiple 24-h recalls for sodium and added sugar measurements rather than biomarkers (e.g., urinary sodium). Diet measurements are always subject to measurement error, and diet among young children can be especially difficult to measure [39]. These study findings may not be generalizable to other US populations, since all of the families were of low SES. We did not have a measure of the child’s stress in order to evaluate the correlation between child stress and externalizing behavior. Finally, there could be unmeasured confounding that we were not able to account for.

## Conclusions

In summary, we found that young children with teacher-reported externalizing behavioral problems had higher proportions of sodium or added sugar in their diet at follow-up in a sex-specific manner. Girls with higher externalizing behavior had higher sodium intake/1000 Calories at follow-up and lower percentage of Calories from added sugar, whereas boys with higher externalizing behavior at baseline had higher percentage of added sugar and lower sodium intake/1000 Calories at follow-up. Further studies are warranted to examine the mechanisms for these associations and to understand the most effective ways to encourage healthy eating habits in boys versus girls with externalizing behaviors. Dietary-based interventions and recommendations directed towards parents/guardians of preschoolers may need to be tailored based on the behavior and sex of the child.

## Additional files

Additional file 1: Table S1A. Top 10 food groups contributing to sodium intake, **Table S1B**. Top 10 food groups contributing to added sugar intake. (DOCX 14 kb)

## Abbreviations

CDC: Center for Disease Control
CES-D: Center for Epidemiologic Studies Depression
GED: General Educational Development
NDSR: Nutrition Data System for Research program
POPS: Preschool Obesity Prevention Series
SCBE: Social Competence and Behavior Scale
SD: Standard deviation
SES: Socioeconomic status
US: United States
USDA: United States Department of Agriculture
