# Supplementary material for: Externalizing behavior is prospectively associated with intake of added sugar and sodium among low socioeconomic status preschoolers in a sex-specific manner
Source: Int J Behav Nutr Phys Act. 2017 Oct 3;14:135. doi: 10.1186/s12966-017-0591-y (PMC5627479; doi:10.1186/s12966-017-0591-y)
Supplement: Supplementary file 1 — Top 10 food groups contributing to sodium intake, Table S1B. Top 10 food groups contributing to added sugar intake. (DOCX 14 kb) [file 12966_2017_591_MOESM1_ESM.docx]

**Supplemental Table 1A**. Top 10 food groups contributing to sodium intake

| Nutrition Data System for Research (NDSR) Food Group | Contribution to total sodium intake, % |
| --- | --- |
| Meat, poultry, and fish recipes | 18.3 |
| Breads, rolls, and other related products | 6.8 |
| Poultry | 6.0 |
| Milk | 5.8 |
| Pasta & Rice | 4.4 |
| Cheese | 4.2 |
| Soup | 3.8 |
| Ready-to-eat cereal | 3.2 |
| Cold cuts and sausage | 2.8 |
| Miscellaneous grain recipes | 2.7 |

**Supplemental Table 1B**. Top 10 food groups contributing to added sugar intake

| Nutrition Data System for Research (NDSR) Food Group | Contribution to total added sugar intake, % |
| --- | --- |
| Soda | 12.0 |
| Milk | 9.9 |
| Fruit juices and drinks | 7.7 |
| Fresh fruit preparations | 6.3 |
| Breads, rolls, biscuits | 5.2 |
| Ready-to-eat cereal | 3.6 |
| Cheese | 3.4 |
| Meat, poultry, and fish recipes | 3.3 |
| Cooked vegetables, fresh, frozen, canned | 3.0 |
| Vegetable recipes | 2.5 |
